# Supplementary material for: Revealing myopathy spectrum: integrating transcriptional and clinical features of human skeletal muscles with varying health conditions
Source: Commun Biol. 2024 Apr 10;7:438. doi: 10.1038/s42003-024-06143-3 (PMC11006663; doi:10.1038/s42003-024-06143-3)
Supplement: Supplementary file 2 — Supplementary Information [file 42003_2024_6143_MOESM2_ESM.pdf]

Supplementary Information

Supplementary Fig. 1. PCA comparison before and after batch adjustment of the integration dataset

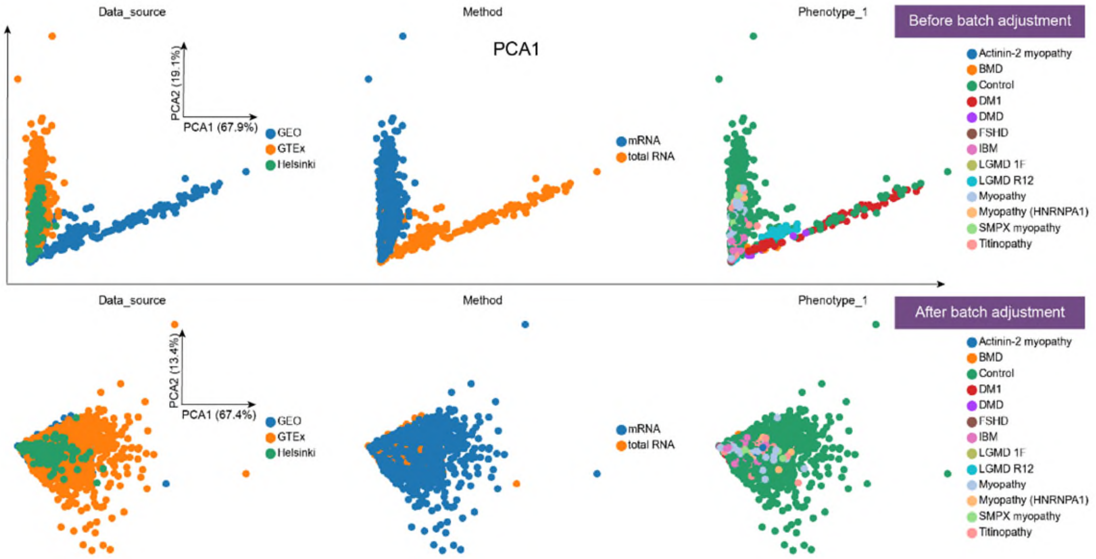

Before batch adjustment, the muscles were mainly clustered based on sequencing method and data sources. After batch adjustment, however, the muscles were basically blended together, regardless of their acquisition methods.

Supplementary Fig. 2 Tissue deconvolution using Tabula Sapiens as reference.

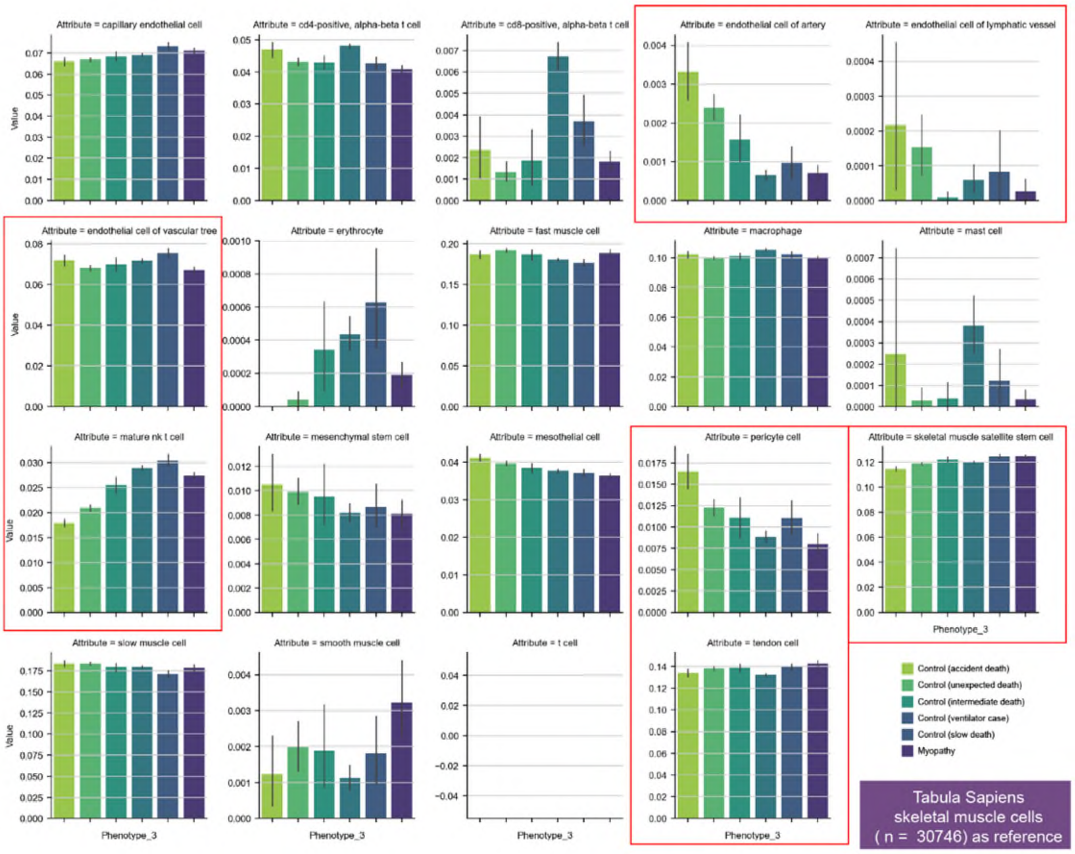

Fewer vasculature structures and more adipocytes and fibroblasts were indicated by myopathy samples.

Supplementary Fig. 3 Tissue deconvolution using GSE143704 as reference.

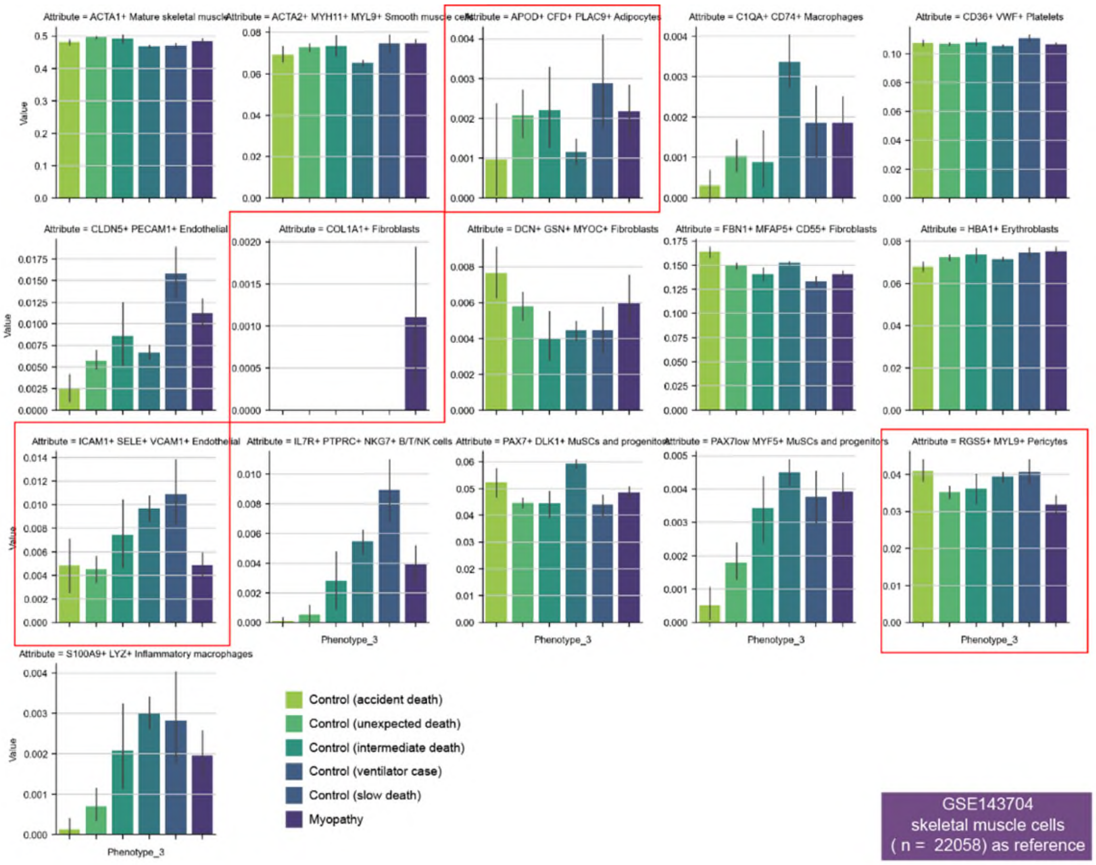

Fewer vasculature structures and more adipocytes and fibroblasts were indicated by myopathy samples.

Supplementary Fig. 4 Pathway analysis in general myopathy

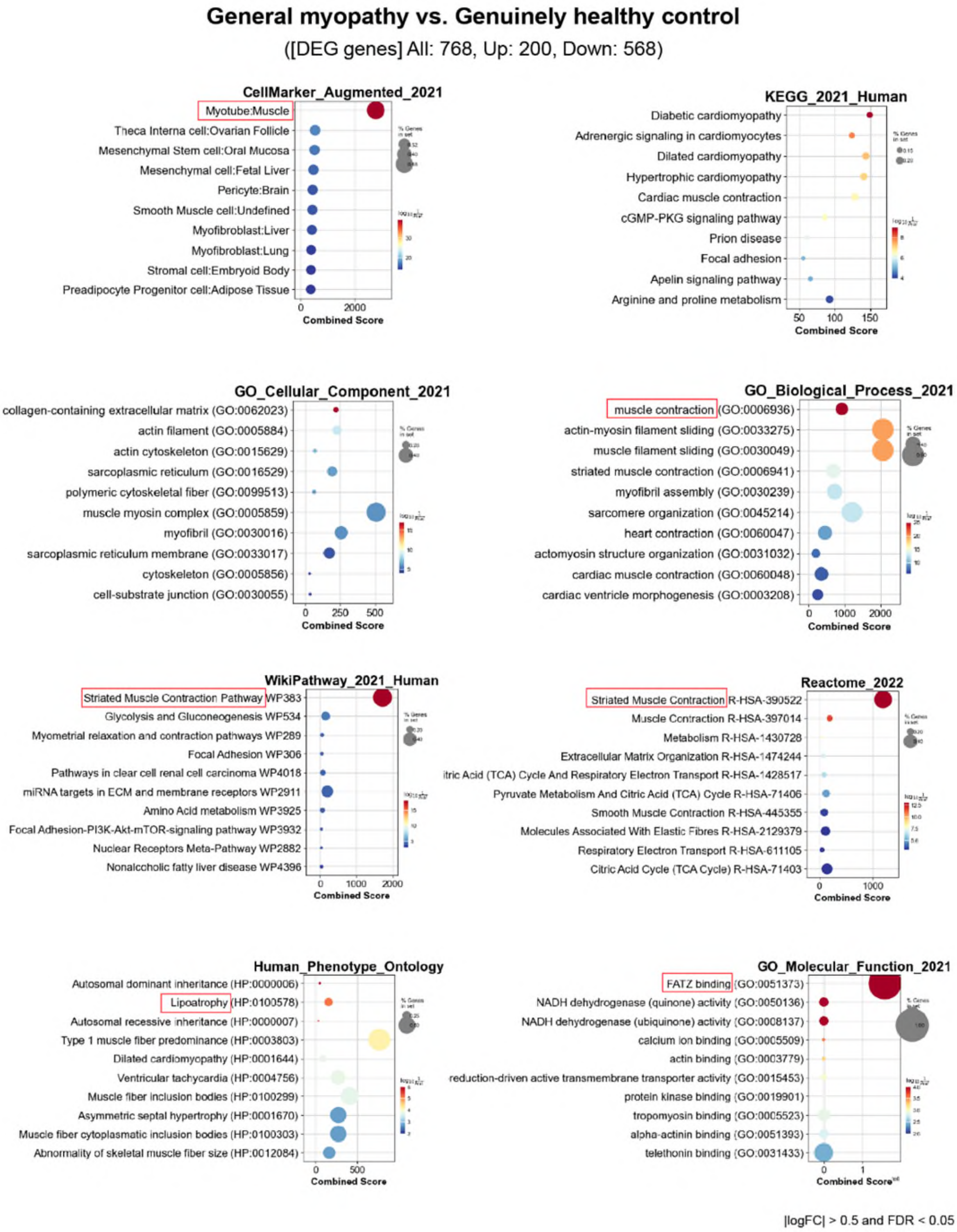

Supplementary Fig. 5 Pathway analysis in congenital myotonic dystrophy

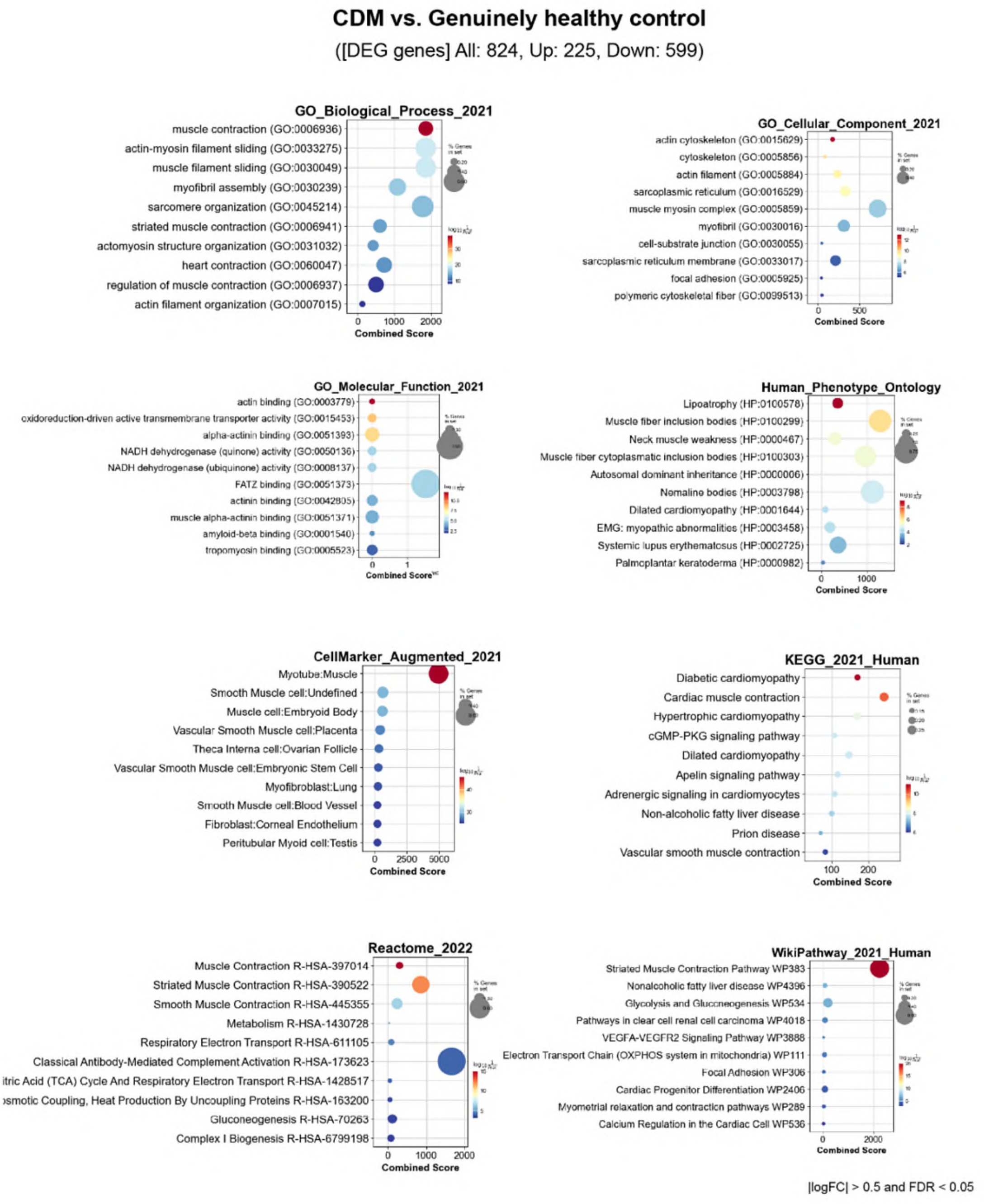

Supplementary Fig. 6 Pathway analysis in myotonic dystrophy type 1

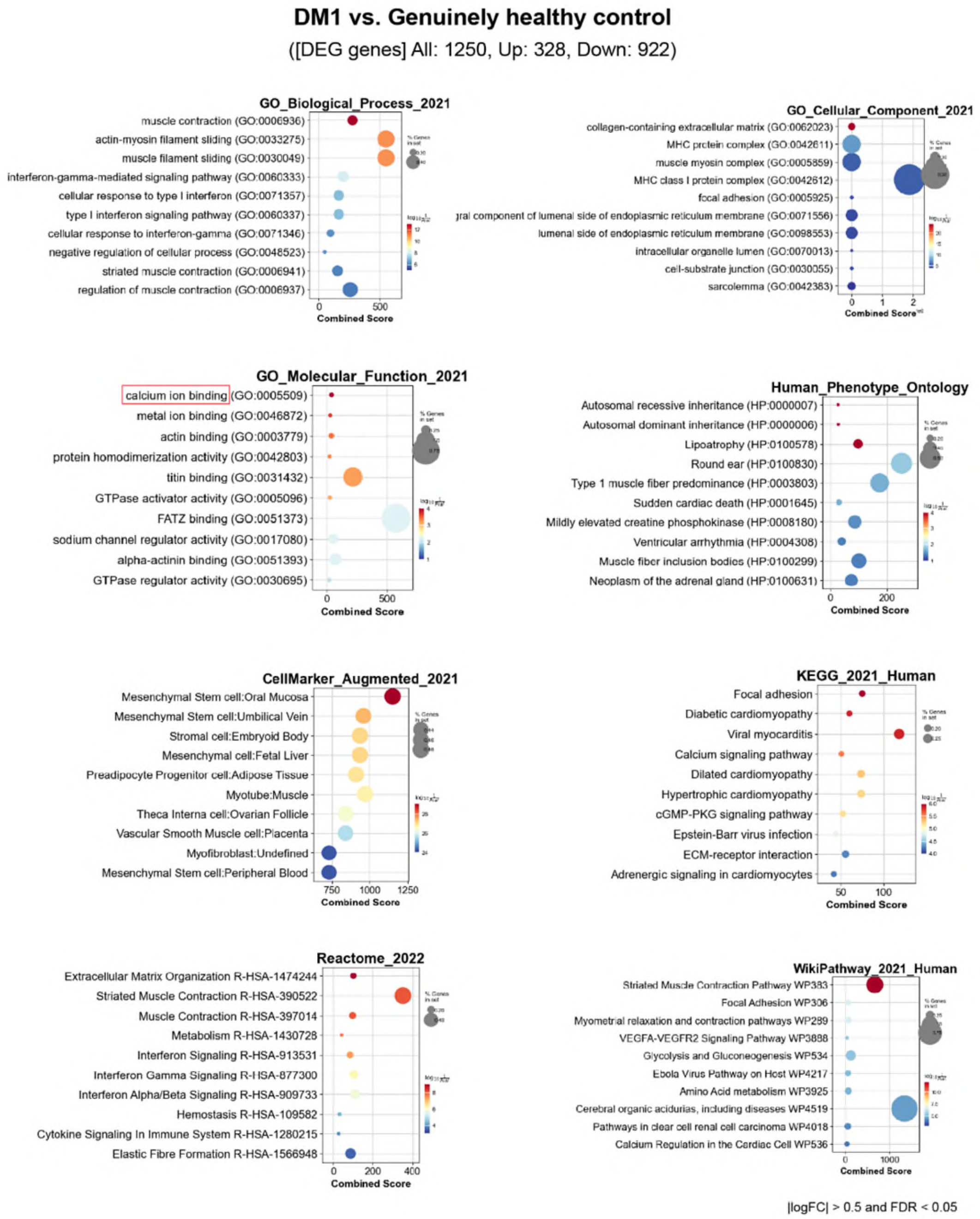

Supplementary Fig. 7 Pathway analysis in facioscapulohumeral muscular dystrophy

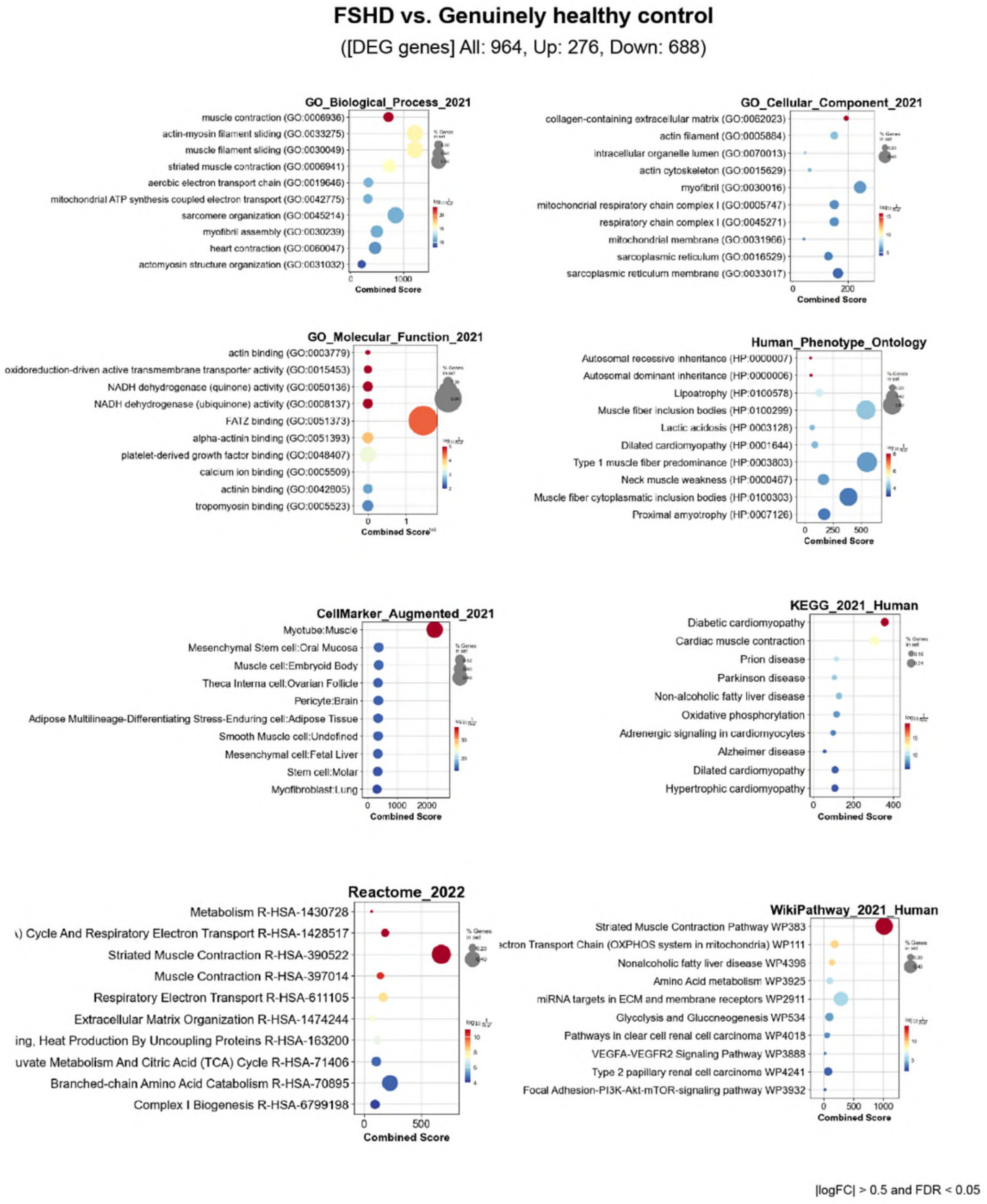

Supplementary Fig. 8 Pathway analysis in inclusion body myositis

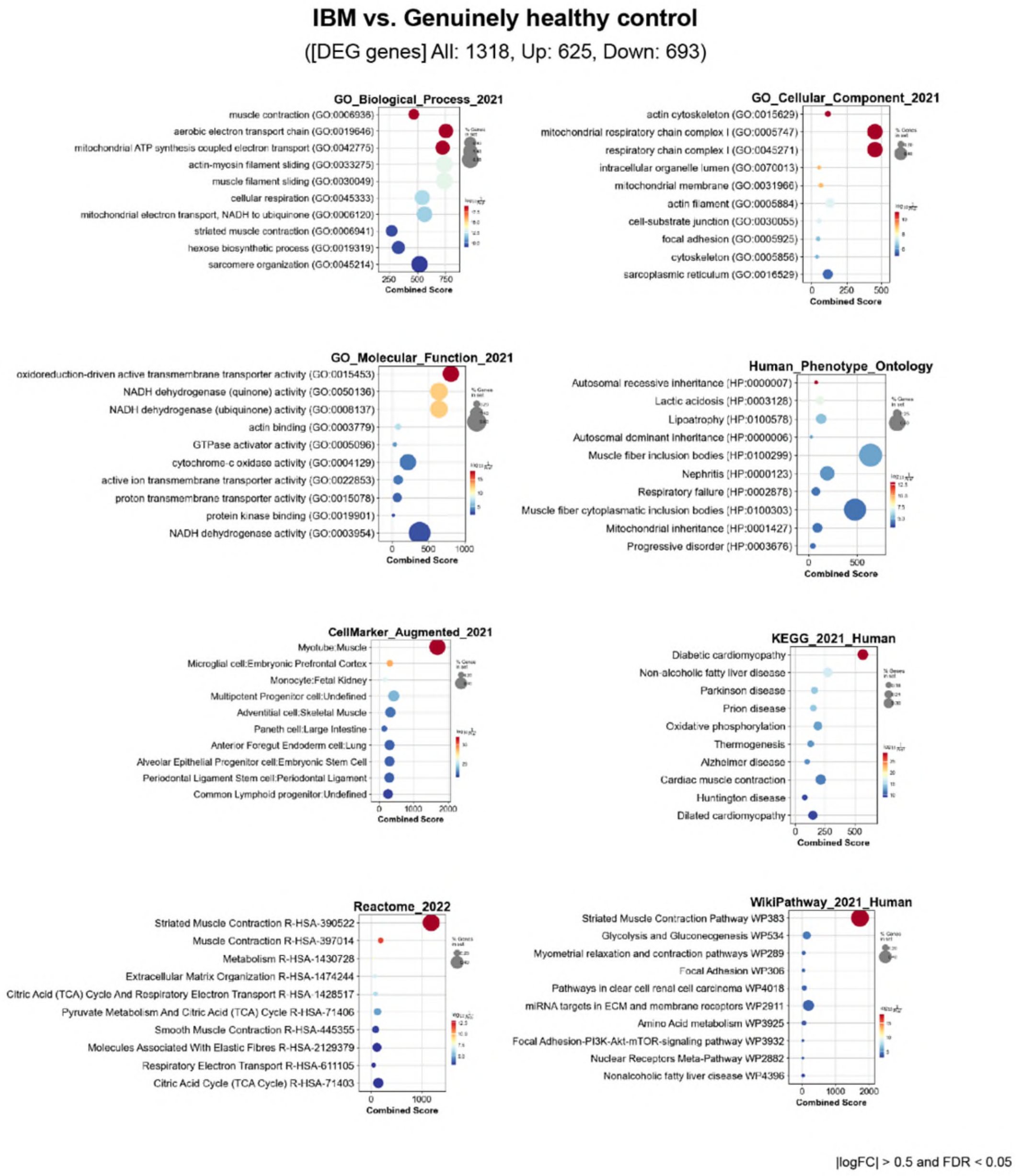

Supplementary Fig. 9 Pathway analysis in limb girdle muscular dystrophies R12

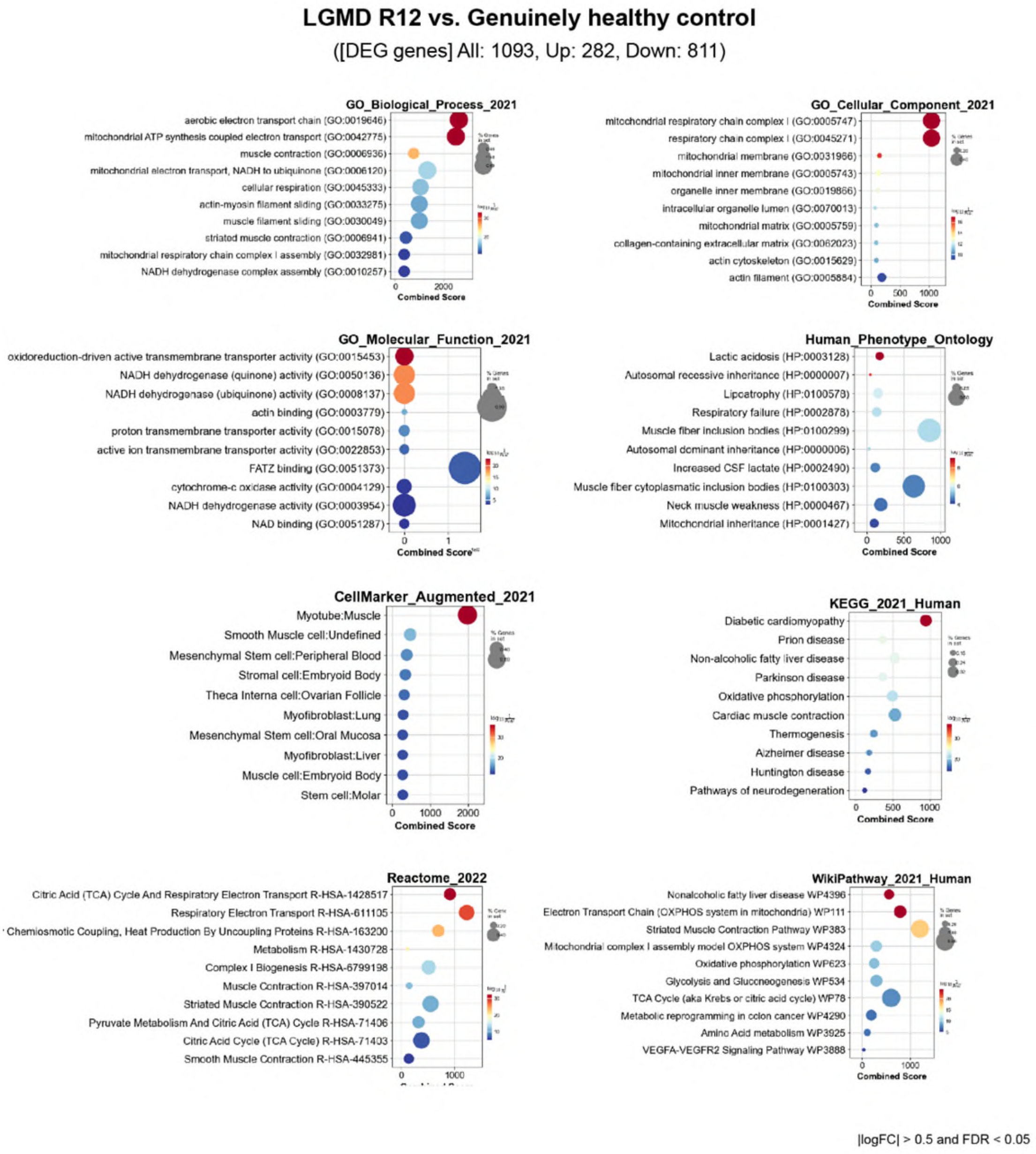

Supplementary Fig. 10 Pathway analysis in titinopathy

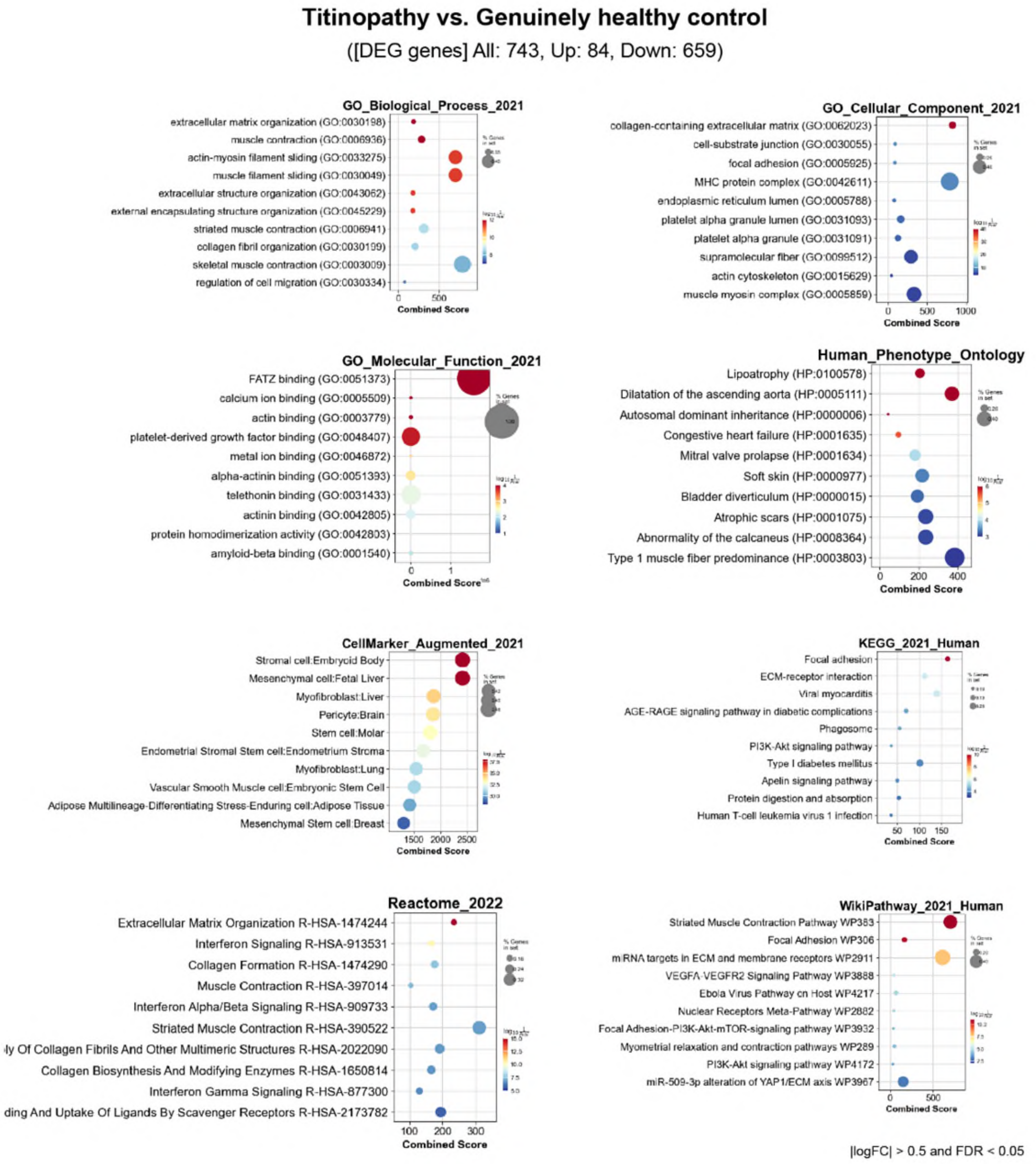

Supplementary Fig. 11 Intersection of the DEG genes between the integration dataset and their results analysed using original data (FSHD, LGMD R12, IBM and CDM).

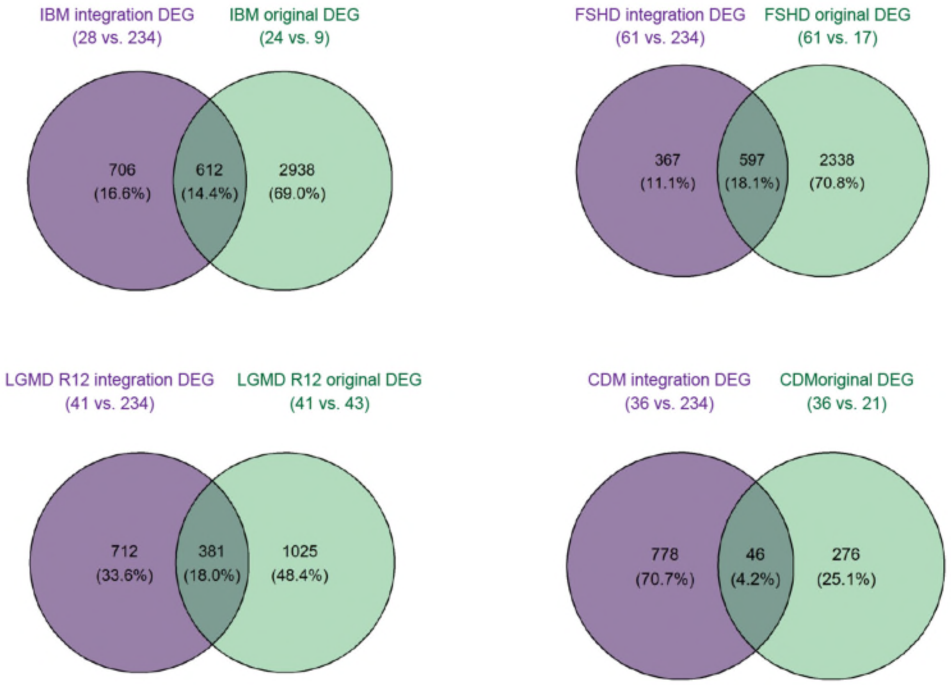

Supplementary Table 1 Primer information for the qPCR validation.

Gene	Sequence
18S_FW<br>18S_RW	5’-CGTCGCTACTACCGATTGGATGG-3’<br>5’-TAATGATCCTTCCGCAGGTTCACCTAC-3’
FASN_FW<br>FASN_RW	5’-AGGGGTGCCTGTCTGAGC-3’<br>5’-CACCATCTTCAGCCCCTG-3’
AOX1_FW<br>AOX1_RW	5’-GGAACCACCCAGAGCCCA-3’<br>5’-ACAGAAAGTCTTGCATGCATCA-3’
CD163_FW<br>CD163_RW	5’-GTGCTACTTGAAGACTCTGGATC-3’<br>5’-ACTTGTTTTCACCATCCACTAGC-3’
CYP4B1_FW<br>CYP4B1_RW	5’-CTTTCTCAAGCTCATCCACCTG-3’<br>5’-CTTTGGCATAGTCAGGCTCATAG-3’
MGST1_FW<br>MGST1_RW	5’-TGACAAGAAAGGTTTTTGCCAAT-3’<br>5’-CTGCGTACACGTTCTACTCTGTCA-3’
PRKCD_FW<br>PRKCD_RW	5’-CTTGAACCAAGTCACCCAGAGAG-3’<br>5’-GGTCTTCTTCTCGAAACCCTGAT-3’

Supplementary Table 2 Top 15 ranked up-regulated FSHD featured genes in the integration dataset and their expression by original RNA count data.

DEG groups	FSHD vs. GTEx genuinely healthy control (61 vs. 234)		FSHD vs. Control (FSHD) (61 vs.17)		FSHD_2019 vs. Control (FSHD_2019) (34 vs.9)		FSHD_2020 vs. Control (FSHD_2020) (27 vs.8)	
**Data type**	**Batch-adjusted read count (Integration dataset)**		**Original read count (Wong et al., 2019 and 2020)**		**Original read count (Wong et al., 2019)**		**Original read count (Wong et al., 2020)**	
**Gene**	**logFC**	**FDR**	**logFC**	**FDR**	**logFC**	**FDR**	**logFC**	**FDR**
HSPA1B	2.230259616	8.11715E-61	0.763163102	0.038642663	0.123084025	0.86937275	1.668154447	0.023948303
MGST1	1.992815829	3.42206E-19	4.215607036	1.98245E-19	4.116693327	1.26536E-09	-1.682350027	0.009837861
HSPA1A	1.644531684	1.86332E-32	0.717992597	0.047517316	0.115900822	0.875343092	1.640654227	0.023654468
FASN	1.550024296	1.00914E-13	2.928102773	6.40511E-09	2.941445866	8.58788E-05	0.508028507	0.603753032
AOX1	1.451110459	5.18737E-19	2.231634861	1.25353E-07	2.181966787	0.00051365	-0.607373492	0.350459537
CD163	1.36154176	3.38631E-15	2.983531031	3.98535E-12	2.86289168	5.10652E-06	-1.064894334	0.08120882
ADH1B	1.357923827	1.65068E-15	1.258968466	0.000225638	1.332870807	0.009488876	0.08131517	0.940708062
PRKCD	1.328097171	5.37449E-23	2.109429085	1.38019E-17	2.307932893	3.45134E-10	-0.631817622	0.100576709
CHRNA1	1.310017597	8.9058E-13	2.548289896	1.72951E-08	2.167699102	0.001012228	-1.546358629	0.02088448
PRKAR2B	1.295598504	8.40959E-09	2.542379161	8.44666E-12	2.444385617	6.66618E-06	-1.193823415	0.031459745
PDE3B	1.272985505	1.86128E-07	3.478194907	3.5089E-11	3.438169864	8.04057E-06	-1.303607326	0.064052441
FADS1	1.240446534	8.9466E-17	1.541173935	0.000244449	1.382254476	0.029062984	-0.702200591	0.287652554
CYP4B1	1.177545954	3.4565E-12	-0.394171308	0.272518401	-0.188624473	0.722834705	1.022045158	0.172766415
GLRX	1.115738971	7.05367E-27	0.456527282	0.1412373	0.312565618	0.533869954	-0.215362196	0.697754425
HMOX1	1.094538606	2.00207E-09	1.558550206	0.002239773	1.525416456	0.049962342	-0.427922615	0.605465091

Supplementary Table 3 Top 15 ranked up-regulated LGMD R12featured genes in the integration dataset and their expression by original RNA count data.

DEG groups	LGMD R12 vs. GTEx genuinely healthy control (41 vs. 234)		LGMD R12 vs. Control (LGMD R12) (41 vs.43)	
Data type	Batch-adjusted read count (Integration dataset)		Original read count (Depuydt et al., 2022)	
Gene	logFC	FDR	logFC	FDR
DPP9	2.201955442	3.8497E-101	1.695428037	3.41399E-31
PDE3B	2.166980329	2.8418E-15	1.971353444	1.32415E-08
PLIN1	1.881998121	1.48683E-08	2.149132803	1.36784E-08
AOX1	1.553765824	7.84971E-16	0.846989507	0.005147695
FASN	1.514758739	5.49803E-10	1.658667664	1.47756E-06
MGST1	1.483594242	3.56553E-08	1.961265134	2.70182E-11
CHRNA1	1.466300989	5.25511E-12	1.360629267	1.75204E-05
CD163	1.460244592	7.62E-13	1.439583692	9.42227E-07
PRKCD	1.429802863	1.02571E-18	1.190667146	2.63148E-11
VSIG4	1.282389425	4.8951E-11	1.249685521	1.79155E-05
FADS1	1.279015303	5.85703E-13	0.781465594	0.012501881
CYP4B1	1.19103035	3.67334E-09	0.23365577	0.467366302
F13A1	1.189991172	8.50872E-08	1.302653881	5.25989E-06
GLRX	1.14896201	5.11399E-21	0.254255877	0.320081988
ERRFI1	1.1248656	1.01284E-11	0.632588934	0.072359258

Supplementary Table 4 Top 15 ranked up-regulated IBM featured genes in the integration dataset and their expression by original RNA count data.

DEG groups	IBM vs. GTEx genuinely healthy control (28 vs. 234)		IBM vs. Control (amputee) (28 vs.24) *		IBM vs. Control (amputee) (24 vs.9)	
Data type	Batch-adjusted read count (Integration dataset)		Original read count		Original read count (Johari et al., 2022)	
Gene	logFC	FDR	logFC	FDR	logFC	FDR
ADH1B	2.283023467	2.16657E-26	1.557356783	2.1238E-06	1.54790287	0.028927638
FASN	2.170599466	1.08998E-15	1.150419127	0.00871562	0.865503908	0.3292392
MGST1	2.077870612	2.59928E-12	3.209737115	4.45646E-16	2.985527665	0.000465459
CD163	1.91502713	4.46575E-17	1.703155676	7.20885E-06	1.809516139	0.001037515
CPM	1.873857945	7.2306E-19	2.229224498	2.61922E-16	2.239747004	0.000198974
AOX1	1.82037842	4.55638E-17	1.112305898	0.00331133	0.870484838	0.171035858
PRKCD	1.803811807	1.19749E-24	1.737721357	1.29753E-14	1.791151405	4.3011E-05
PYGL	1.803348464	1.99256E-16	2.473661498	1.92228E-20	2.470601366	6.18485E-06
FABP4	1.798701076	7.62896E-13	3.00638345	6.42224E-20	3.048061583	0.000296283
FADS1	1.795477103	8.50173E-20	1.311639362	0.000890676	0.947267376	0.061051256
C1QB	1.669063994	2.81253E-10	3.196945514	6.02985E-16	3.160250829	2.32845E-07
F13A1	1.637839759	5.08086E-11	2.573979467	9.72368E-12	2.367276587	4.0104E-05

VSIG4	1.59669872	4.16659E-13	1.82380994	9.62339E-07	1.775546114	0.000346693
CPAMD8	1.586373517	3.6824E-12	1.364228184	1.36562E-06	1.372513317	0.07245495
AGPAT2	1.471251706	4.34552E-14	1.943679928	8.92894E-17	1.754561485	0.002268381

* This DEG groups is an updated re-analysis of our previous work (Johari et al., 2022) with enlarged samples from our local muscles.

Supplementary Table 5 Top 15 ranked up-regulated CDM featured genes in the integration dataset and their expression by original RNA count data.

**DEG groups**	**CDM vs. GTEx genuinely healthy control (36 vs. 234)**		**CDM vs. Control (pediatric) (36 vs.21)**	
**Data type**	**Batch-adjusted read count (Integration dataset)**		**Original read count (Hale et al., 2022)**	
**Gene**	**logFC**	**FDR**	**logFC**	**FDR**
PRKCD	1.247857078	2.18735E-13	0.072907312	0.890060931
VSIG4	1.242126715	1.63869E-09	-0.091693593	0.870421533
MGST1	1.225025587	2.48198E-05	0.689671148	0.307073346
LYVE1	1.19432704	1.25202E-08	0.740619103	0.101835769
CD163	1.175767126	9.17196E-08	-0.210227579	0.700652136
KRT10	1.163946287	6.12364E-15	1.555460086	1.58273E-07
CHRNA1	1.154988064	7.49036E-07	1.037943808	0.036124659
AOX1	1.073391294	3.10779E-07	-0.286463245	0.55933502
IGFBP3	1.047960224	4.1737E-08	-0.734967869	0.049315661
C1QB	1.022005857	6.58454E-05	0.177451243	0.807154683
TKT	0.974245167	2.36278E-08	-0.083156449	0.858442414
CNTFR	0.954840249	3.66774E-10	0.264448251	0.452379261
ADH1B	0.945105174	2.04659E-05	-0.602787167	0.181118546
F13A1	0.941911664	0.000102676	0.153754577	0.825064916
FADS1	0.93844913	1.09147E-06	-0.606310394	0.111992323
