# Supplementary material for: Revealing myopathy spectrum: integrating transcriptional and clinical features of human skeletal muscles with varying health conditions
Source: Commun Biol. 2024 Apr 10;7:438. doi: 10.1038/s42003-024-06143-3 (PMC11006663; doi:10.1038/s42003-024-06143-3)
Supplement: Supplementary file 3 — Description of Additional Supplementary Files [file 42003_2024_6143_MOESM3_ESM.pdf]

Description of Additional Supplementary Files

File name: Supplementary Data 1
Description: Meta data for the integration dataset.

File name: Supplementary Data 2
Description: Differential expression analysis results in general myopathy and six specific myopathies.

File name: Supplementary Data 3
Description: qPCR validation results.
