# Supplementary material for: Revealing myopathy spectrum: integrating transcriptional and clinical features of human skeletal muscles with varying health conditions
Source: Commun Biol. 2024 Apr 10;7:438. doi: 10.1038/s42003-024-06143-3 (PMC11006663; doi:10.1038/s42003-024-06143-3)
Supplement: Supplementary file 7 — Reporting Summary [file 42003_2024_6143_MOESM7_ESM.pdf]

nature portfolio

Corresponding author(s): Huahua Zhong

Last updated by author(s): Feb 26, 2024

Reporting Summary

Nature Portfolio wishes to improve the reproducibility of the work that we publish. This form provides structure for consistency and transparency in reporting. For further information on Nature Portfolio policies, see our Editorial Policies and the Editorial Policy Checklist.

Statistics

For all statistical analyses, confirm that the following items are present in the figure legend, table legend, main text, or Methods section.

n/a	Confirmed	
☐	☒	The exact sample size (*n*) for each experimental group/condition, given as a discrete number and unit of measurement
☐	☒	A statement on whether measurements were taken from distinct samples or whether the same sample was measured repeatedly
☐	☒	The statistical test(s) used AND whether they are one- or two-sided<br>*Only common tests should be described solely by name; describe more complex techniques in the Methods section.*
☐	☒	A description of all covariates tested
☒	☐	A description of any assumptions or corrections, such as tests of normality and adjustment for multiple comparisons
☐	☒	A full description of the statistical parameters including central tendency (e.g. means) or other basic estimates (e.g. regression coefficient) AND variation (e.g. standard deviation) or associated estimates of uncertainty (e.g. confidence intervals)
☐	☒	For null hypothesis testing, the test statistic (e.g. *F*, *t*, *r*) with confidence intervals, effect sizes, degrees of freedom and *P* value noted<br>*Give P values as exact values whenever suitable.*
☒	☐	For Bayesian analysis, information on the choice of priors and Markov chain Monte Carlo settings
☐	☒	For hierarchical and complex designs, identification of the appropriate level for tests and full reporting of outcomes
☒	☐	Estimates of effect sizes (e.g. Cohen's *d*, Pearson's *r*), indicating how they were calculated

Our web collection on statistics for biologists contains articles on many of the points above.

Software and code

Policy information about availability of computer code

Data collection	The data sources include 803 muscles from the GTEx Consortium (dbGaP Accession phs000424.v8.p2),11 291 muscles from the GEO database (GSE115650,12 GSE140261,13 GSE175861,14 GSE184951,15 GSE201255,16 GSE20274517), and 127 muscles from Helsinki (39 of which have also been reported as GSE15175718)
Data analysis	Python (version 3.8.1) and R (version 4.2.2) were utilized to analyze the data. The integrated raw data counts were adjusted using a negative-binomial-regression-based batch effect adjustment tool, ComBat-seq (for bulk RNA-seq count data), and a normalization algorithm, Trimmed Mean of M-values (TMM) (https://gitlab.com/georgy.m/conorm), which is presumably better for between-sample comparisons.20,21 To decrease the impact of low-expression genes, a simple and stringent filtering rule was applied to every sample: muscle-specific gene counts should be > 0 in all samples (n = 1221) and 9231 genes were selected from 16953 candidates. The visualization of the integrated dataset was conducted using a single-cell analysis pipeline (Scanpy), which includes principal component analysis (PCA) and uniform manifold approximation and projection (UMAP) analyses.22 The data and code used for this study are publicly available at GitHub (https://github.com/Hirririiir/Myopathy_spectrum).

For manuscripts utilizing custom algorithms or software that are central to the research but not yet described in published literature, software must be made available to editors and reviewers. We strongly encourage code deposition in a community repository (e.g. GitHub). See the Nature Portfolio guidelines for submitting code & software for further information.

Data

Policy information about availability of data

All manuscripts must include a data availability statement. This statement should provide the following information, where applicable:

- Accession codes, unique identifiers, or web links for publicly available datasets
- A description of any restrictions on data availability
- For clinical datasets or third party data, please ensure that the statement adheres to our policy

The analyzing scripts and the integration dataset are available in the GitHub (https://github.com/Hirririiir/Myopathy-Spectrum). The original data of each public datasets used in this study are available in their original research (links are also provided in the Myopathy-Spectrum GitHub repository).

Research involving human participants, their data, or biological material

Policy information about studies with human participants or human data. See also policy information about sex, gender (identity/presentation), and sexual orientation and race, ethnicity and racism.

Reporting on sex and gender	The female to male ratio is 363:737 in the integration dataset, and the sex information is not available for 121 donors.
Reporting on race, ethnicity, or other socially relevant groupings	Though detailed ethnicity information is not available for most muscle donors, the GTEx and GEO datasets are mainly composed of Caucasians, and the Helsinki dataset is mainly composed of Finns.
Population characteristics	The control groups included muscles from 929 controls: accident death control (n = 31), unexpected death control (n = 203), intermediate death control (n = 46), ventilator case control (n = 424), slow death control (n = 87), other controls (n = 111), amputee control (n = 24), and hyperCkemia control (n = 3). The female to male ratio is 363:737, and the sex information is not available for 121 donors. The age range is as follows: 0-9 years (n = 46), 10-19 years (n = 20), 20-29 years (n = 83), 30-39 years (n = 105), 40-49 years (n = 165), 50-59 years (n = 303), 60-89 years (n = 389). Age information is not available for 119 donors.
Recruitment	The donors of the GTEx and GEO datasets were sourced from their original studies, the donors of the Helsinki dataset were recruited from Tampere Neuromuscular Center (Finland).
Ethics oversight	The included studies had been approved by each of their ethics committees.

Note that full information on the approval of the study protocol must also be provided in the manuscript.

Field-specific reporting

Please select the one below that is the best fit for your research. If you are not sure, read the appropriate sections before making your selection.

☒ Life sciences ☐ Behavioural & social sciences ☐ Ecological, evolutionary & environmental sciences

For a reference copy of the document with all sections, see nature.com/documents/nr-reporting-summary-flat.pdf

Life sciences study design

All studies must disclose on these points even when the disclosure is negative.

Sample size	This is a retrospective observational study, sample size calculation is not applicable.
Data exclusions	The inclusion and exclusion criteria for participant selection were as follows: 1) only human skeletal muscle tissue was included (no cell lines or organoids); 2) bulk-RNA sequencing was performed using high-throughput techniques (no chip arrays or single-cell data); 3) datasets were preserved in raw count format (those shared in transformed count format were excluded).
Replication	Clinical information have been used to validate the myopathy spectrum order, and quantitative PCR was applied in our local muscles (Helsinki dataset) to validate some featured genes identified by the myopathy Spectrum
Randomization	This is a retrospective observational study, randomization is not applicable.
Blinding	This is a retrospective observational study, blinding is not applicable.

Reporting for specific materials, systems and methods

We require information from authors about some types of materials, experimental systems and methods used in many studies. Here, indicate whether each material, system or method listed is relevant to your study. If you are not sure if a list item applies to your research, read the appropriate section before selecting a response.

Materials & experimental systems

n/a	Involved in the study	
☒	☐	Antibodies
☒	☐	Eukaryotic cell lines
☒	☐	Palaeontology and archaeology
☒	☐	Animals and other organisms
☐	☒	Clinical data
☒	☐	Dual use research of concern
☒	☐	Plants

Methods

n/a	Involved in the study	
☒	☐	ChIP-seq
☒	☐	Flow cytometry
☒	☐	MRI-based neuroimaging

Clinical data

Policy information about clinical studies

All manuscripts should comply with the ICMJE guidelines for publication of clinical research and a completed CONSORT checklist must be included with all submissions.

Clinical trial registration	This is a retrospective observational study, there is no clinical trial registration number.
Study protocol	Retrospective observational study
Data collection	Integrative analysis (database + local data)
Outcomes	Myopathy severity Inferred by sequenced bulk RNA-seq data.
